# Effect of inhibiting prolactin secretion on secondary hair follicle development in cashmere goats

**DOI:** 10.5713/ab.25.0053

**Published:** 2025-05-12

**Authors:** Chunhui Duan, Xuejiao Yin, Kexing Hao, Lechao Zhang, Yuchun Xie, Xueru Li, Yueqin Liu, Yingjie Zhang

**Affiliations:** 1 College of Animal Science and Technology, Hebei Agricultural University, Baoding, China; 2 College of Animal Science and Technology, Hebei Normal University of Science and Technology, Qinhuangdao, China; 3 Hebei Key Laboratory of Specialty Animal Germplasm Resources Exploration and Innovation, Qinhuangdao, China

**Keywords:** Fos, Goat, Hair Follicle, Hair Papilla Cells, Kit, Prolactin

## Abstract

**Objective:**

This study aims to investigate the molecular mechanisms by which inhibiting prolactin (PRL) secretion affects secondary hair follicle (SHF) development in cashmere goats.

**Methods:**

A total of 20 cashmere goats were randomly assigned to either a bromocriptine (PRL inhibitor, BCT, 0.06 mg/kg BW) treatment (BCT+) or a control (BCT−) group. Blood and skin samples were collected monthly during telogen, and cashmere samples were collected after grow. Furthermore, the dermal papilla cells (DPCs) were isolated from SHF and cultured with PRL.

**Results:**

The results showed that the percentage of active SHF in telogen decreased (p<0.05) in the BCT+ group. The inhibition of PRL secretion reduced (p<0.05) the serum PRL concentration, and the expression of the PRL, SPRLR, Kit, and Fos genes. Transcriptome analysis of skin tissues identified differentially expressed genes. The results of the *in vitro* experiment indicated that 150 ng/mL PRL promoted (p<0.05) the proliferation and migration of DPCs.

**Conclusion:**

The Kit gene mediates PRL’s regulation of SHF activation by stimulating the activation of Fos. These findings demonstrate that inhibiting PRL secretion in telogen can reduce the number of activated SHFs and width of hair bulbs.

## INTRODUCTION

Hair follicle growth is cyclic and can be divided into anagen, catagen, and telogen, which are regulated by follicular mesenchymal and epithelial cell populations, especially mesenchymal dermal papilla cells (DPCs) [1,2]. The transition of hair follicles from telogen to anagen involves various signaling pathways such as the shh, Wnt/β-catenin/Lef-1, and STAT3 [3–5]. Prolactin (PRL) is one hormone involved in cashmere growth regulation. Previous studies have shown that PRL is associated with fleece and cashmere growth in sheep and goats, respectively [6,7]. It was shown that 200 and 400 μg/L of PRL promoted the growth of secondary hair follicles (SHFs) and fibers of cashmere goats in anagen, whereas 50 μg/L PRL had no effect [8]. Subcutaneous injection of exogenous PRL increased SHF activity during telogen, resulting in early shedding of cashmere, while dopamine receptor agonists reduced serum PRL levels, delayed primary hair follicle (PHF) and SHF reactivation [9], and increased cashmere production [10]. Thus, high levels of PRL can be seen as an activation signal for SHF in telogen, prompting SHF to enter the cashmere growth phase, while inhibiting active SHFs duting anagen. PRL requires binding to its receptor (PRLR) to provide its physiological function. Bignon et al [11] cloned sequences of sheep PRL long-type receptor (L-PRLR) and short-type receptor (S-PRLR), finding that both were also present in goats. Although both receptor types have similar hormone-binding activities, they activate different signaling pathways [12].

Currently, there are only a few studies on how PRL regulates SHF activation in cashmere goats. The mechanisms underlying the cyclic growth of hair follicles remain unclear. Bromocriptine (BCT) is a kind of dopamine receptor agonist, which tends to bind to the D2 receptor in the pituitary gland. After the D2 receptor is activated, it reduces the calcium level of cells through the G13 protein, thus inhibiting the release of PRL [13]. This study used high-throughput sequencing of skin tissues to explore the relationship between PRL and SHF activation in cashmere goats, and to screen related differential expressed genes. The SHF DPCs were isolated to study the effect of PRL on their proliferation of SHF growth DPCs and its mechanism. The overexpression and interference vectors of related genes were constructed to detect their effects on the proliferation and apoptosis of DPCs, and to reveal the role of PRL in promoting the growth and development of hair follicles.

## MATERIALS AND METHODS

### Experimental animals

This experiment was performed at the Qinglong Lihong Cashmere Goat Farm located in the Qinhuangdao Province of China.

After uniform shearing of cashmere goats, 20 healthy Yanshan cashmere goats (6 months old, male) with similar body weights were selected. All goats were maintained under natural photoperiod and had free access to feed and water. Dietary information has been reported in our previous research [14].

Twenty cashmere goats were randomly divided into 2 groups: control (BCT−, n = 10) group (non-treated group), and treatment (BCT+, n = 10) group, treated daily with 0.06 mg/kg BW [9] of PRL inhibitor (BCT), which was supplied as tablets (Bromocriptine Mesilate Tablets; Gedeon Richter, Budapest, Hungary). The tablets were dissolved in water and then sprayed onto the concentrate feed for the experimental period (2 months).

### Sample collection

Blood samples were collected by jugular venipuncture of each goat into 5 mL coagulation-promoting tubes before feeding in the morning on days 30 and 60 after BCT treatment. The blood samples were immediately centrifuged at 1,000 ×g for 15 min and were stored at −20°C until analysis.

Skin samples were taken from above the left scapula region of each goat on the same occasion. One of the skin samples was soaked in a bottle containing Bouin’s fixative for 24 h and the other skin sample was immediately placed in liquid nitrogen. The right scapular hair fibers were collected at the end of the experiment and stored in sealed bags. The daylight hours and maximum and minimum temperatures during the experimental period are shown in Supplement 1.

The stretched length of the cashmere fibers was measured to the nearest millimeter of each cashmere. These measurements were conducted using 50 fibers randomly selected from the fleece sample of each goat [15]. The diameter of over 2,000 randomly chosen fibers was measured using an Automatic Fiber Diameter Analyzer (BEION F10, Shanghai, China).

### Serum measurements

Serum concentrations of PRL (0.5 ng/mL–200 ng/mL, CV <10%) and GH (0.05 ng/mL–20 ng/mL, CV<10%) were analyzed using commercial Goat ELISA kits (Nan-jing Jiancheng Bioengineering, Nanjing, China) according to the manufacturer’s instructions.

### Transcriptome sequencing

Three skin samples were randomly chosen from each experimental group as the treatment (T1, T2, and T3) and control (C1, C2, and C3) before being subjected to high-throughput sequencing technology (Novogene, Beijing, China). Total RNA was extracted by Trizol Up (Invitrogen, Carlsbad, CA, USA) and detected using an Agilent 2100 bioanalyzer (Agilent, Santa Clara, CA, USA). Library construction, reference genome ( https://www.ncbi.nlm.nih.gov/search/all/?term=txid9925) assembly, and Illumina sequencing were conducted. Differential genes were analyzed by DESeq2 analysis (−1>log2FC>1, FDR<0.05). Cluster Profiler performed differentially expressed gene (DEG) GO and KEGG enrichment analyses. STRING ( https://cn.string-db.org/) analyzed the protein interaction network of DEGs, and a protein interaction score >0.15 was used as a screening condition to obtain protein–protein interaction (PPI) information.

### Histopathology and immunofluorescence

Skin tissue sections were prepared according to the methods mentioned in our previous study [14]. The number of PHFs and SHFs, SF-to-PF ratio, percentage of active PHF, and active SHF (PASF) were analyzed in transverse sections. Longitudinal sections were counted for epidermal thickness, primary/secondary follicle depth (PFD/SFD), and primary/secondary hair bulb width (PFBW/SFBW).

Paraffin longitudinal sections were subjected to Kit and Fos protein immuno-histochemistry. Antigen repair buffer (pH 6.0) (Servicebio, Wuhan, China) was used for antigen repair, and 3% BSA was used for 30 min at room temperature. The primary antibody (1:200, rabbit anti-c-kit and rabbit anti-cfos, Bioss, Beijing, China) was incubated overnight at 4°C. The secondary antibody (1:200, HRP-labeled goat antirabbit secondary antibody, Servicebio) was incubated for 50 min at room temperature. The nuclei were restained with hematoxylin (Servicebio); then, the images were acquired, and the optical density values of the SHFs were detected by ImageJ software.

### Cell culture and treatment

Small excised parts of the body side skin were depilated, washed, and sterilized with 75% alcohol. Then, remove some obvious fat and connective tissue on the surface, cut into 1 mm 2 tissue blocks, collect in a culture dish containing 0.25% neutral protease for incubation. Separate the dermis and subcutaneous tissue under a stereomicroscope, expose the hair follicle bulge, and gently pull out the hair bulb of the bulge in the opposite direction of hair follicle growth. Collect the cell in a centrifuge tube, when a sufficient amount is collected, digest with type IV collagenase, wash, pass through a cell sieve, and inoculate in a culture flask for culture.

DPCs were purified by using the sensitivity of DPCs to different digestive enzymes and differential adhesion. When the DPCs grew to a good monolayer, they were digested with 0.25% trypsin and then gently blown on the cell-attached bottle wall. The cell suspension was discarded, then collagenase D was added for digestion, and the medium was added to blow into the cell suspension. The suspension of DPCs was inoculated into a new culture flask, incubated at 37°C for about 2 h, and the unattached cells were discarded by gently shaking the culture flask. The adherent cells were pure goat DPCs. DPC was identified by α-SMA immunofluorescence. The purity of DPC cells isolated by this method can reach more than 98 % DPCs at the second passage grown on coverslips were washed and fixed in 4% paraformaldehyde for 15 min at 4 °C after being treated with 0.2% Triton X-100 for 10 min. The DPCs were blocked with 2% BSA for 30 min. Cells were incubated overnight with a mouse anti-α-SMA antibody (1:200, Abcam, Burgess Hill, UK) at 4°C and with a secondary antibody (1:400, Abcam) at 37°C for 1 h. After DAPI staining, images were obtained via fluorescence microscopy.

DPCs were seeded in 12-well plates and culture was continued until cell confluence reached 80% for further studies. Cells were treated with PRL (150 ng/mL), combined with PRL and Anta-PRLR (1,500 ng/mL), or treated with Anta-PRL alone. Anta-PRLR, a PRL receptor antagonist, was generated by substituting glycine at position 129 with arginine (G129R). This mutation abolishes its agonistic activity while retaining receptor-binding capability, enabling competitive binding to PRLR and blocking endogenous PRL from engaging the receptor. Cells were collected 12 h after treatment for follow-up index determination.

### Cell proliferation assay

DPCs at the second passage were seeded into 96-well culture plates at 5,000 cells/well after 0, 50, 150, 200, and 400 ng/mL of PRL was added (Prospec, East Brunswick, NJ, USA). The number of cells in the 4 wells was counted at each time point (24 h, 48 h, and 72 h, respectively), and the averages were used to plot the cell growth curve. Each experiment was repeated three times.

### Cell migration determination

DPCs at the 3rd passage were seeded into 6-well plates at 1.0×10^6^ cells/well. The DMEM/F12 medium (serum-free) for culturing the DPCs in the 6-well plates was supplemented with PRL solutions (diluted in PBS) at final concentrations of 150 ng/mL. Equal volumes of PBS were added to the control group instead of the PRL solutions. Cell migration analysis was performed using the *in vitro* scratch assay method.

### Plasmid construction and transfection

The mRNA sequence of the Kit gene in goats was downloaded from the NCBI, which was synthesized by the company Golden Viz. NheI/XbaI digestion sites were added and cloned into the vector pGWLV10-new (GENEWIZ, Cambridge, MA, USA); then, the over-expression vector Kit-pGWLV10-new (o-Kit) was constructed. The shRNA lentivirus was prepared by Jinweizhi Company (Table 1). In simple terms, it involves co-transfecting the lentiviral vector containing the target gene with two packaging plasmids into HEK293T cells. After transfection, the cell culture medium was collected at 48 and 72 h. The virus was concentrated by ultracentrifugation or using commercial virus concentration kits to increase the viral titer.

A 96-well plate was inoculated with 1.0×10^4^ cells/well and incubated for 24 h. After lentiviral encapsulation of the target plasmid, the titer (TU/mL, represents the number of functional transduction units per milliliter of virus solution, reflecting the actual infection efficiency of the virus.) was calculated based on the number of cells, infection efficiency, and amount of virus added. Subsequently, the cells were infected with lentivirus (MOI: 5, 50, 100, 150, 300) in DMEM/F12 medium supplemented with 4% FBS and 8 μg/mL polybrene. After an additional incubation period of 72 h, GFP expression was evaluated using microscopy.

### Western blot analysis

DPCs in the o-Kit and sh1-Kit groups were washed twice with PBS and lysed by RIPA (Servicebio) for 3 min to collect total protein, and BCA (Servicebio) was used to determine the protein concentration. After denaturation, the protein was transferred to PVDF (0.45 μm) (Servicebio) membrane by SDS-PAGE method. The primary antibody (1:1,000, Servicebio) was added and shaken overnight at 4°C. Then, the secondary antibody (1:5,000, Servicebio), was added at room temperature for 30 min. After ECL (Servicebio) hypersensitive luminescence processing, the images were used Alpha (Alpha Innotech, San Leandro, CA, USA) for optical density values analysis.

### Reverse transcription quantitative polymerase chain reaction

Reverse transcription quantitative polymerase chain reaction (RT-qPCR) was used to detect the mRNA expression levels of the *Kit*, *Fos*, *FGF21*, *DUSP1*, *HSPA6*, *PRL*, *L-PRLR*, *SPRLR*, *Bax*, *Bcl-2*, and *Caspase-3* genes. The primer information is provided in Table 2. The RT-qPCR method was the same as our previous studies [14].

### Statistical analysis

All statistical analyses were carried out using SPSS 21.0 software (IBM, Armonk, NY, USA). Statistical differences were compared using unpaired two-tailed Student’s t test or ANOVA. All experiments were repeated at least in triplicate. p< 0.05 was considered to indicate statistical significance. Data are expressed as mean±standard deviation.

## RESULTS

### Prolactin affects the development of secondary hair follicle

**Figure 1:**
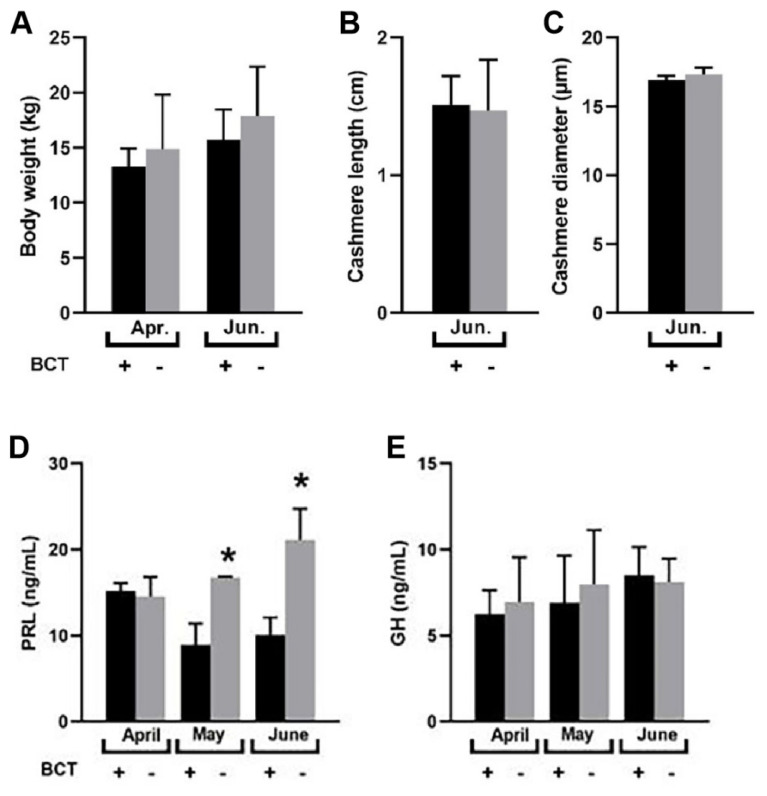
Prolactin (PRL) affects the development of SHF. Body weight (A), cashmere length (B), and cashmere diameter (C) in BCT+ (treated with bromocriptine, n = 10) and BCT− (control, n = 10) groups. Serum concentrations of PRL (D) and GH (E) in treatment (BCT+, n = 10) and control (BCT−, n = 10) groups. Data are expressed as mean±SD. * Indicates significant difference (p<0.05). SHF, secondary hair follicle; SD, standard deviation.

Inhibition of PRL secretion did not (p>0.05) affect body weight, newborn cashmere length, or diameter (Figures 1A–1C) but reduced (p<0.05) serum PRL levels (Figure 1D) without affecting (p>0.05) GH levels during telogens (Figure 1E).

The inhibition of PRL secretion reduced (p = 0.022 and p<0.001, respectively) the percentage of active secondary hair follicles (PASF) and the SFBW during the experiment period. In addition, there was an interaction (p = 0.010 and p<0.001, Table 3) between the month and treatment. After 30 days of treatment, the PRL inhibitor-treated goats (BCT+) showed lower (p<0.05, Table 4) PASF, SFBW, and SFD compared to the control group (BCT−).

### Transcriptome sequencing

**Figure 2:**
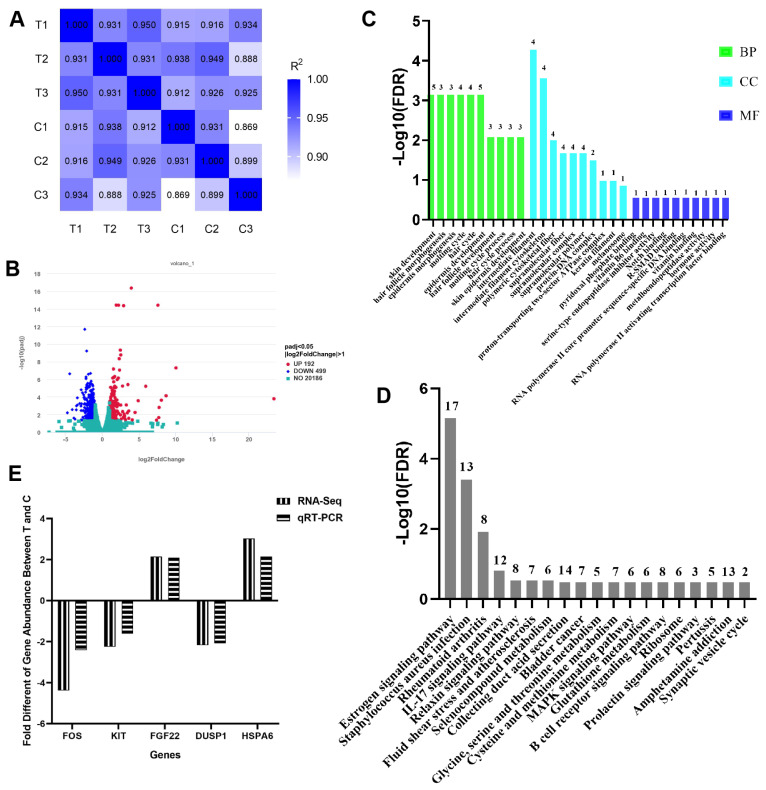
Transcriptome sequencing. Person correlation (A) of skins and volcano map (B) of DEGs. T1, T2, and T3: the skin tissues from the BCT+ group. C1, C2, and C3: skin tissues from BCT− group. (C) GO enrichment of DEGs from skin. Top 10 GO terms in the BP, CC, and MF categories. (D) KEGG enrichment of DEGs from skin. Top 20 KEGG pathways. (E) qPCR validation of five DEGs with biological replicates. BP, biological process; CC, cellular component; MF, molecular function; RT-qPCR, reverse transcription quantitative polymerase chain reaction; DEGs, differentially expressed genes.

The quality of RNA from the skin tissues of cashmere goats met the requirements for library construction (Supplement 2). There were 41,280,479 reads in the BCT+ group and 41,643,848 reads in the BCT− group, which could be compared with the goat genome (Supplement 3). A total of 22,690 functionally annotated goat protein-coding genes were obtained based on Fragments Per Kilobase of exon model per Million mapped fragments (FPKM) values (FPKM>0), of which 21,419 were from the BCT+ group and 22,163 from the BCT− group. The collagen alpha 1 chain (COL1A1) was the most abundantly expressed in both groups (FPKM> 180,000) (Supplement 4). There was a high correlation between the samples (R^2^>0.86) in accordance with the experiment replication requirement (Figure 2A). There were 691 DEGs (FDR<0.05) (Figure 2B), of which 192 genes were up-regulated, and 499 genes were down-regulated in the BCT+ group, respectively. The top 10 up-regulated differential genes and down-regulated differential genes are shown in Supplement 5.

Based on the screening criteria of GO function enrichment FDR<0.05 (Figure 2C), a total of 82 significantly enriched GO entries were screened, including 63 entries for the biological process (BP), 3 entries for the cellular component (CC), and 16 entries for molecular function (MF). The top GO Directed Acyclic Graph (DAG) analysis revealed that the final summaries in BP were skin development (GO: 0043588), epithelial cell migration (GO: 0010631), and vascular development (GO: 0001944) (Supplement 1A). CC eventually aggregated into the stromal membrane (GO: 0009929) (Supplement 1B). The DEGs in MF were finally aggregated to regulate RNA polymerase II-related activity (GO:0000981, GO:0000977, GO:0000987) (Supplement 1C). The top 20 most significant pathways are shown in Supplement 6. The KEGG enrichment pathways (Figure 2D) involved in PRL and the most significantly differential Fos gene were selected to map the gene network interactions (Supplement 7). The expression of the PDGFB, SOS1, Kit, RELN, CREB5, Fos, DUSP1, and ELK4 genes were all down-regulated in the BCT+ group, in which Kit was upstream of Fos and interacted with both PRLR and Fos (Supplements 8, 9). This indicated that the inhibition of PRL secretion during the SHF activation phase resulted in the down-regulation of PRL downstream-signaling-related factors that may play an important role in SHF activation. The RT-qPCR results showed similar trends for five differential genes with RNA-Seq results (Figure 2E), including Kit and Fos.

PPI analysis (Supplement 10) of DEGs revealed 17 proteins interacting with both PRLR and Fos. Among these, PDGFB showed the highest PRLR interaction score, while NR3C1 had the strongest Fos association. Pathway analysis identified significant MAPK enrichment (FDR<0.05) only for PDGFB, IGF2, and Kit. GO term enrichment revealed distinct functional roles for PDGFB (29 terms; epithelial migration, vascular development), IGF2 (8 terms; skin/muscle development, cell proliferation), and Kit (18 terms; epithelial motility, angiogenesis) (Supplement 9). These data suggest PRLR, Fos, PDGFB, IGF2, and Kit coordinate PRL-mediated secondary follicle activation in cashmere goats via MAPK-dependent and context-specific mechanisms.

### Immunohistochemistry of skin tissue

**Figure 3:**
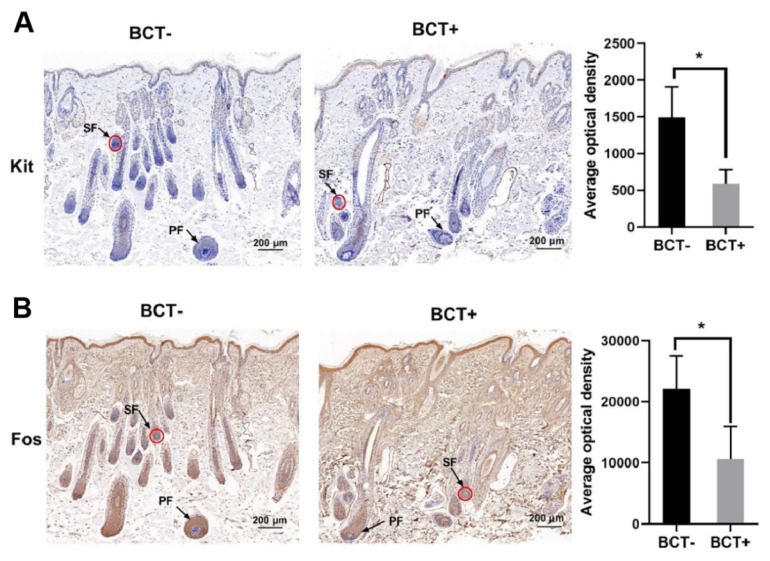
Immunohistochemistry (IHC) of Fos and Kit in SHFs. (A) Kit IHC and average optical density value. (B) Fos IHC and average optical density value. * Indicates significant difference in the BCT+ and BCT− groups (p<0.05). BCT+, goats treated with PRL inhibitor; BCT−, control group; PF, number of primary hair follicles; SF, number of secondary hair follicles; SHF, secondary hair follicle; PRL, prolactin.

Kit and Fos were positively expressed in the bulbs of SHFs. We detected the optical density of the SHFs bulb by ImageJ software. The Kit showed focal brownish-yellow staining (Figure 3A), and Fos showed massive brownish-yellow staining (Figure 3B). The protein expression of both Kit and Fos in the BCT+ group were lower (p<0.05) than those of the BCT− group.

### Prolactin promoted the proliferation and migration of dermal papilla cells

**Figure 4:**
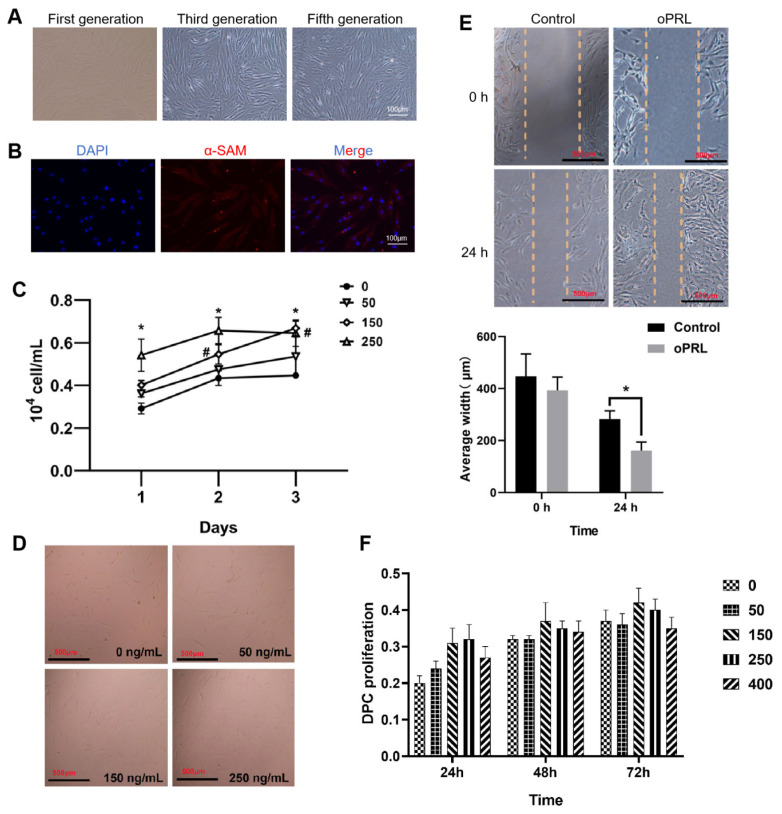
Effect of PRL on DPC proliferation and migration. (A) First-generation, third-generation DPCs and fifth-generation DPCs. (B) α-SAM identification of isolated SF DPCs. (C) Counting of DPCs treated with different concentrations of PRL. (D) DPCs treated with different concentrations of PRL for 48 h. (E) The 150 ng/mL PRL-treated DPCs ‘average scratch width. (F) Effect of different PRL concentrations on DPC proliferation. *, ^#^ Indicate significant difference between the treatment, and control group (p<0.05). PRL, prolactin; DPC, dermal papilla cell.

The isolated and cultured DPCs of SHF exhibited multilayered aggregated growth, with increased aggregation towards the growth center. As cell spacing widened, DPCs enlarged and changed shape from triangular to long spindle-like, resembling fibroblasts. DPCs cultured up to the third and fifth generations were also observed in a distinct cellular state with normal growth and proliferation (Figure 4A). Positive α-SMA expression (red fluorescence) and nuclear DAPI staining confirmed these isolated cells as SHF DPCs (Figure 4B).

The addition of 150 ng/mL and 250 ng/mL PRL promoted (p<0.05) the proliferation of DPCs (Figure 4F), which was consistent with the results of the cell count (p<0.05, Figures 4C, 4D). In addition, 150 ng/mL PRL could promote (p<0.05) DPC migration (Figure 4E).

### Effect of prolactin on mRNA expression of dermal papilla cell-related genes

**Figure 5:**
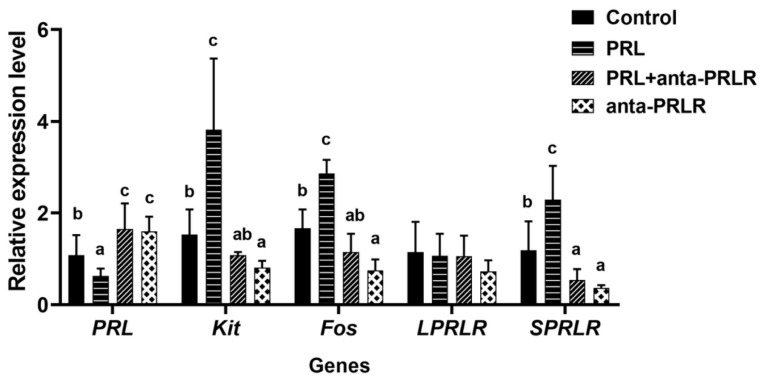
Relative expression of genes associated with PRL and anta-PRLR treated in hair papilla cells. Data are expressed as mean±SD. ^a–c^ Values within a row with different superscripts differ significantly at p<0.05. PRL, prolactin; SD, standard deviation.

The effect of PRL on the mRNA expression of DPC-related genes is shown in Figure 5. The addition of 150 ng/mL PRL increased (p<0.05) Kit, Fos, SPRLR, and IGF-1 mRNA expression levels and decreased (p<0.05) PRL mRNA expression levels in DPCs. Compared with 1,500 ng/mL anta-PRLR, gene expression levels were opposite (p<0.05) after adding PRL or PRL+anta-PRLR .

The sh1-Kit, sh2-Kit, and sh3-Kit vectors were successfully constructed (Supplement 11A). After lentiviral encapsulation of the target plasmid, the titer was calculated based on the number of cells, infection efficiency, and amount of virus added. The virus titers of o-Kit, sh1-Kit, sh2-Kit, and sh3-Kit were 3.67×10^8^ TU/mL, 2.33×10^8^ TU/mL, 2.67×10^8^ TU/mL, and 2.13×10^8^ TU/mL, respectively. TU/mL represents the number of functional transduction units per milliliter of virus solution, reflecting the actual infection efficiency of the virus. The highest infection efficiency was observed at an MOI value of 300 for infected viruses according to GFP fluorescence (Supplement 11B). Therefore, DPCs were infected with lentivirus encapsulated with Kit over-expression and an interference vector with an MOI value of 300.

### Expression of Kit, Fos, and apoptotic factors in lentivirus infected dermal papilla cells

**Figure 6:**
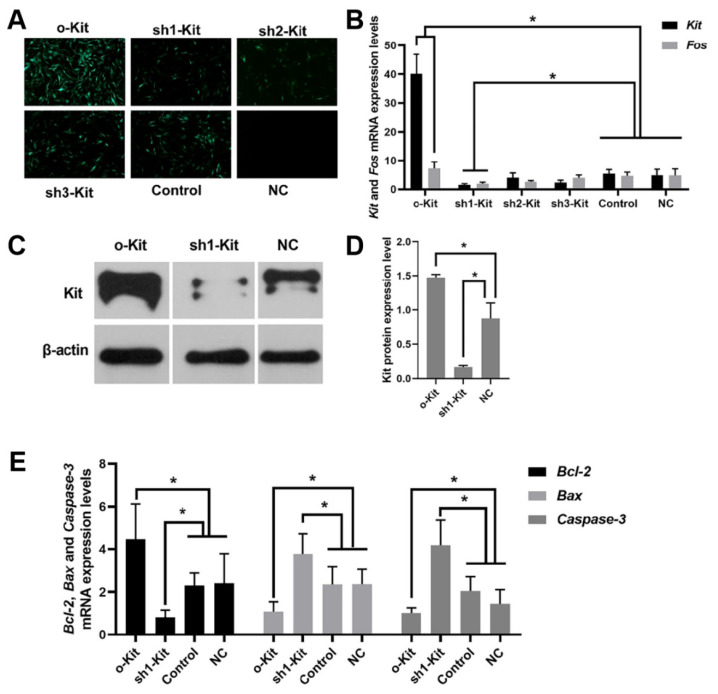
Lentiviral infection of DPCs regulated apoptosis factors’ mRNA expression. (A) Fluorescence of lentiviral-infected (MOI = 300) DPCs. (B) Kit and Fos mRNA expression levels. (C, D) Kit protein expression levels. (E) Bax, Bcl-2, and Caspase-3 mRNA expression levels. Control indicates empty vehicle; NC indicates no plasmid added. * Indicates a significant difference between the treatment and control groups (p<0.05).

Fluorescence images showed more cells in the o-Kit group than others, with no fluorescence in the blank control (Figure 6A). RT-qPCR analysis revealed that, relative to the NC group, the expression levels of Kit and Fos genes were increased (p<0.05) in the o-Kit group, whereas they were substantially reduced (p<0.05) in the sh1 group (Figure 6B).

Compared with the NC group, the Kit protein expression level was up-regulated (p<0.05) in the o-Kit group and down-regulated (p<0.05) in the sh1-Kit group (Figures 6C, 6D).

The Bax and Caspase-3 genes had the lowest expression levels in the o-Kit group and highest expression levels in the sh1-Kit group, which was significantly different (p<0.05) from the other groups. Bcl-2 gene expression was the highest in the o-Kit group and lowest in the sh1-Kit group, which was significantly different (p<0.05) from the other groups (Figure 6E).

## DISCUSSION

In this study, inhibiting PRL secretion during the telogen phase did not affect the cashmere growth cycle, suggesting that PRL inhibition during this phase does not influence the subsequent growth of cashmere fibers. Previous studies indicated that subcutaneous injection of exogenous PRL during the cashmere non-growing period leading to early shedding, and subcutaneous injection of dopamine receptor agonists delays PHF and SHF reactivation in cashmere goats [9], and increases cashmere production [10]. Discrepancies in PRL response may be due to treatment timing, breed, and geography. In this study, BCT was treated from late telogen to early anagen, which was probably the main reason for not affect the cashmere fibers growth and diameter in the next cycle of cashmere growth.

The hair follicle is a relatively complex morphological structure of the skin appendage, which is the origin of hair, controls hair growth, and has a unique structure and cyclic regenerative capacity [16]. At the termination of the hair follicle cycle, the matrix cells stop proliferating, and the lower part of the follicle undergoes degeneration [17]. With the renewal of SHF growth, substantial proliferative activity occurs at the beginning of the next growth cycle. Studies have found that the inhibition of PRL secretion during the cashmere growing period increased SHF activity and had no effect on PHF activity, while PHF and SHF underwent a rapid growing and non-growing growing phase process after inhibition was eliminated [18]. In this study, we found that low PRL levels during the follicle activation period inhibit SHF development, while high levels of PRL facilitate hair follicle activation.

Increased serum PRL concentration during early cashmere growth accelerates shedding and SHF rebuilding before the next growth cycle [19,20]. We previously reported that inhibiting of serum PRL secretion in the cashmere growing period increased SHF activity without affecting PHF activity [14]. The above results demonstrated that reducing the serum PRL levels during the cashmere growing period in Yanshan cashmere goats may prolong its duration, and reducing PRL during the cashmere non-growing period delayed the transition of SHF to the cashmere growing period.

DPCs can secrete a diverse array of growth factors and regulators to modulate the activity of follicular stem cells, promote the proliferation and differentiation of follicular keratinocytes, and thereby orchestrate the growth and development of hair follicles [21]. In this study, PRL was found to increase the proliferation and migration of DPCs, inhibit the early apoptosis of DPCs, and suppress the rate of late apoptosis or necrosis of DPCs. Anagen is the process of downward hair follicle growth and development regulated by mesenchymal cell signaling, in which DPCs play an essential role [22]. We found that the secondary hair bulbs in the BCT+ group were significantly smaller than those in the BCT− group, indicating that the high concentration of PRL during the activation period supports the development of hair bulbs. The presence of DPCs in the hair bulb suggests that PRL may influence hair follicle development by regulating dermal papilla development. The addition of PRL down-regulated PRL mRNA expression, did not change L-PRLR mRNA expression in DPCs, and up-regulated SPRLR mRNA expression, which suggested that SPRLR played a vital role in the regulation of DPC proliferation by PRL.

Vascular endothelial growth factor (VEGF), an autocrine growth factor for DPCs, enhances their proliferation and migration [23,24]. Moreover, the down-regulation of the PF4 gene increased the VEGF gene expression level and promoted DPC growth [25], while high concentration of PF4 inhibited human hair follicle growth by down-regulating the IGF-1 expression level [26]. In this study, we found that PRL promoted the mRNA expression levels of VEGF and IGF-1 while decreasing the PF4, suggesting that PRL may promote the proliferation of DPCs through VEGF.

The Fos gene generally exists in the nucleus and can act on the gene-free encoding nuclear protein as a key gene involved in early cell proliferation; it does not express in telogen. Under the influence of various factors, if the cell rapidly shifts to the proliferative phase, the Fos protein can act by producing trans-acting factors; then, it can be involved in the regulation of cell proliferation and signaling by producing trans-acting factors if the cells undergo a rapid transition to proliferation [27]. In the present study, the PPI network revealed that the Fos protein interacted with the PRLR protein; meanwhile, the Fos mRNA expression levels were up-regulated in the PRL group. *In vivo* transcriptome analysis revealed a decrease in Fos mRNA expression level and protein levels in skin tissue after the inhibition of PRL secretion, demonstrating that Fos is involved in PRL regulation of DPC proliferation and apoptosis.

Many studies on the Kit gene have tended to focus on the hair color formation of mammalians, and SCF/Kit signaling plays an essential role in maintaining melanocyte formation, maturation, proliferation, and differentiation, as well as migration [28]. DPCs can express and secrete SCF [29]. Therefore, Kit may be involved in PRL regulation of Fos expression, affecting the growth of DPCs. To test this, we constructed Kit gene lentiviral over-expression and interference vectors and infected DPCs in cashmere goats. We found that the Fos gene was up-regulated in the Kit over-expression group and down-regulated in the interference group. Moreover, the expression of proapoptotic genes Bax and Caspase-3 was lower in the o-Kit group than in the interference group, and the expression of the anti-apoptotic gene Bcl-2 gene was higher in the o-Kit group than in the interference group. Our results indicate that the Kit gene is involved in regulating DPC proliferation by PRL through the Fos gene.

## CONCLUSION

In summary, PRL stimulates cellular proliferation and migration while inhibiting apoptosis by up-regulating Kit expression. Furthermore, suppressing PRL secretion during the non-cashmere growth phase can reduce the number of activated hair follicles and the width of hair bulbs. Additionally, the genes SPRLR, Fos, Kit, PDGFB, and IGF2 play important roles in the PRL-mediated activation of SHFs.

## Figures and Tables

**Table 1:** shRNA fragments of Kit genes

Name	shRNA sequence (5′–3′)
Kit-shRNA-1-F	CCGGTGCAATTACACGTGCACCAATACTCGAGTATTGGTGCACGTGTAATTGC
Kit-shRNA-1-R	CGCGTGCAATTACACGTGCACCAATACTCGAGTATTGGTGCACGTGTAATTGC
Kit-shRNA-2-F	CCGGTGCAAGTCCATGCTGTCAAAGACTCGAGTCTTTGACAGCATGGACTTGC
Kit-shRNA-2-R	CGCGTGCAAGTCCATGCTGTCAAAGACTCGAGTCTTTGACAGCATGGACTTGC
Kit-shRNA-3-F	CCGGTGCACAGACGAAGAAGAATAGCCTCGAGGCTATTCTTCTTCGTCTGTGC
Kit-shRNA-3-R	CGCGTGCACAGACGAAGAAGAATAGCCTCGAGGCTATTCTTCTTCGTCTGTGC

**Table 2:** Primer information

Gene	GenBank accession No.	Sequence (5′-3′)	Product (bp)	Efficiency (%)	Tm (°C)
*FOS*	XM_005686096.3	F: GAGTCGGCGCATTACAGAGA	184	97.4	60
		R: ACACACTCCATGCGTTTTGC			
*Kit*	XM_018049156.1	F: CACGCTGGTTCGCTGT	86	96.5	60
		R: GCCCTTCTGATTCGCT			
*FGF22*	XM_013965510	F: GTGGGCGTCGTGGTGC	212	99.9	60
		R: GGTGTCGCTGTGTGCG			
*DUSP1*	XM_018065720.1	F: AAGAACGCAGGACGAC	150	98.2	60
		R: TGAAAGCGAAGAAGGA			
*HSPA6*	XM_05677146.3	F: ACCCCGTAACAGAAAT	126	97.2	60
		R: GAGAAAAACCGAAAGC			
*PRL*	100861193	F: TCCTGGAGCCAAAGAGACTG	81	96.8	60
		R: TGACGTGCCTCTTCATCCTT			
*L-PRLR*	100861318	F: CTCAGGCCTATCCCTCCAAG	61	98.7	60
		R: TCGGGATTCTCCAGCTTCTC			
*SPRLR*	GU075814.1	F: GCAGTGGCTTTGAAGGGCTAT	113	99.0	60
		R: AGGCGAGAAGGCTGTGAT			
*Bax*	XM_013971446.2	F: AACATGGAGCTGCAGAGGAT	208	100.5	60
		R: CCAATGTCCAGCCCATGATG			
*Caspase-3*	XM_018041755.1	F: TGGACCCGTCGATCTGAAAA	195	96.4	60
		R: GCGTACAAGAAGTCTGCCTC			
*Bcl-2*	XM_018039337.1	F: TCTTTGAGTTCGGAGGGGTC	263	95.9	60
		R: GGAGAAATCAAACAGGGGCC			
*β-ACTIN*	102179831	F: CCCTGGAGAAGAGCTACGAG	98	99.2	60
		R: CAGGAAGGAAGGCTGGAAGA			

**Table 3:** Effect of inhibition of PRL secretion on hair follicle traits in cashmere goats

Traits^1)^	Group		Month			p-value		

	BCT−	BCT+	April	May	June	Month	Group	Month× Group
PF (mm^2^)	2.78±0.62	2.82±0.85	3.37±0.68^b^	2.40±0.57^a^	2.62±0.34^a^	<0.001	0.884	0.651
SF (mm^2^)	25.99±6.70	26.55±5.59	26.01±7.73	26.39±5.80	26.38±4.66	0.691	0.491	0.636
S/P	10.54±3.96	10.01±3.26	9.00±4.27^a^	11.44±3.92^b^	10.37±1.75^b^	<0.001	0.539	0.610
PAPF (%)	0.47±0.21	0.51±0.19	0.56±0.25^b^	0.44±0.20^a^	0.46±0.11^a^	0.007	0.193	0.293
PASF (%)	0.47±0.12^b^	0.43±0.13^a^	0.36±0.11^a^	0.50±0.15^b^	0.48±0.07^b^	<0.001	0.022	0.010
ET (μm)	20.37±3.71	18.49±3.70	19.39±3.67	18.75±5.17	20.14±1.86	0.205	0.067	0.573
PFD (μm)	1,422.07±127.80	1,399.75±107.11	1,459.71±112.36	1,402.25±124.69	1,370.78±101.89	0.058	0.492	0.912
SFD (μm)	962.81±81.56	934.18±130.67	899.19±87.99^a^	896.95±92.91^a^	1,049.34±66.54^b^	<0.001	0.101	0.011
PFBW (μm)	123.85±24.23	117.03±18.39	123.51±16.87	122.23±29.61	115.57±15.78	0.512	0.295	0.516
SFBW (μm)	57.31±10.72^b^	53.45±11.05^a^	43.88±3.03^a^	54.35±6.69^b^	67.90±3.81^c^	<0.001	<0.001	<0.001

Data are expressed as mean±SD.

1) BCT−: control group; BCT+: goats treated with prolactin (PRL) inhibitor.

a–c The values within a row with different superscripts differ significantly at p<0.05.

PF, number of primary hair follicles; SF, number of secondary hair follicles; S/P, number of secondary hair follicles/number of primary hair follicles; PAPF, percentage of active primary hair follicles; PASF, percentage of active secondary hair follicles; ET, epidermal thickness; PFD, primary hair follicle depth; SFD, secondary hair follicle depth; PFBW, primary hair bulb width; SFBW, secondary hair bulb width; SD, standard deviation.

**Table 4:** PASF, SFD, and SFBW of cashmere goats in different months

Traits^1)^	April		May		June	

	BCT−	BCT+	BCT−	BCT+	BCT−	BCT+
PASF (%)	0.35±0.02^a^	0.38±0.02^a^	0.54±0.02^c^	0.42±0.02^ab^	0.57±0.02^c^	0.46±0.02^bc^
SFD (μm)	928.21±79.30^b^	870.16±90.46^ab^	941.36±85.45^b^	852.54±80.88^a^	1,018.85±50.17^c^	1,079.83±69.02^c^
SFBW (μm)	43.52±2.01^a^	44.24±3.88^a^	61.25±1.14^c^	51.51±4.83^b^	67.46±4.16^d^	62.34±9.58^cd^

Data are expressed as mean±SD.

1) BCT−: control group; BCT+: goats treated with prolactin (PRL) inhibitor.

a–d Values within a row with different superscripts differ significantly at p<0.05.

PASF, percentage of active secondary hair follicles; SFD, secondary hair follicle depth; SFBW, secondary hair bulb width; SD, standard deviation.

## SUPPLEMENTARY MATERIAL

**Supplement 1**. GO enrichment of DEGs from skin.

**Supplement 2**. RNA quality from cashmere goat skin.

**Supplement 3**. Statistics result of RNA-Seq reads comparison from skin.

**Supplement 4**. Top 20 mRNAs expressed in skin samples.

**Supplement 5**. Top 10 up- and downregulated differential genes from skin.

**Supplement 6**. Top 20 pathways of KEGG enrichment.

**Supplement 7**. KEGG enrichment of DEGs from skin.

**Supplement 8**. PRLR and Fos protein network interactions.

**Supplement 9**. DEG information for interactions with PRLR and Fos proteins.

**Supplement 10**. Protein network interactions of DEGs from skin in cashmere non-growing period.

**Supplement 11**. Screening for optimal MOI value for infected DPCs.

## References

[1] Ito N, Sugawara K, Bodó E (2010). Corticotropin-releasing hormone stimulates the in situ generation of mast cells from precursors in the human hair follicle mesenchyme. J Invest Dermatol.

[2] Sennett R, Rendl M (2012). Mesenchymal–epithelial interactions during hair follicle morphogenesis and cycling. Semin Cell Dev Biol.

[3] Sano S, Kira M, Takagi S, Yoshikawa K, Takeda J, Itami S (2000). Two distinct signaling pathways in hair cycle induction: stat3-dependent and -independent pathways. Proc Natl Acad Sci USA.

[4] Alonso L, Fuchs E (2006). The hair cycle. J Cell Sci.

[5] Huelsken J, Vogel R, Erdmann B, Cotsarelis G, Birchmeier W (2001). β-Catenin controls hair follicle morphogenesis and stem cell differentiation in the skin. Cell.

[6] Choy VJ, Nixon AJ, Pearson AJ (1997). Distribution of prolactin receptor immunoreactivity in ovine skin and changes during the wool follicle growth cycle. J Endocrinol.

[7] Winder LM, Scobie DR, Bray AR, Bickerstaffe R (1995). Wool growth rate in vitro is independent of host animal nutrition, season, and the potential mediators of photoperiod, melatonin and prolactin. J Exp Zool.

[8] Ibraheem M, Galbraith H, Scaife JR, Ewen S (1994). Growth of secondary hair follicles of the cashmere goat in vitro and their response to prolactin and melatonin. J Anat.

[9] Dicks P, Russel AJF, Lincoln GA (1994). The role of prolactin in the reactivation of hair follicles in relation to moulting in cashmere goats. J Endocrinol.

[10] Wuliji T, Litherland A, Goetsch AL (2003). Evaluation of melatonin and bromocryptine administration in Spanish goats. Small Rumin Res.

[11] Bignon C, Binart N, Ormandy C, Schuler LA, Kelly PA, Dijane J (1997). Long and short forms of the ovine prolactin receptor: cDNA cloning and genomic analysis reveal that the two forms arise by different alternative splicing mechanisms in ruminants and in rodents. J Mol Endocrinol.

[12] Nixon AJ, Ford CA, Wildermoth JE, Craven AJ, Ashby MG, Pearson AJ (2002). Regulation of prolactin receptor expression in ovine skin in relation to circulating prolactin and wool follicle growth status. J Endocrinol.

[13] Giraldi EA, Ioachimescu AG (2020). The role of dopamine agonists in pituitary adenomas. Endocrinol Metab Clin North Am.

[14] Zhang L, Duan C, Guo Y, Zhang Y, Liu Y (2021). Inhibition of prolactin promotes secondary skin follicle activation in cashmere goats. J Anim Sci.

[15] Duan C, Xu J, Sun C, Jia Z, Zhang W (2015). Effects of melatonin implantation on cashmere yield, fibre characteristics, duration of cashmere growth as well as growth and reproductive performance of inner Mongolian cashmere goats. J Anim Sci Biotechnol.

[16] Roh C, Tao Q, Lyle S (2004). Dermal papilla-induced hair differentiation of adult epithelial stem cells from human skin. Physiol Genom.

[17] Parry AL, Nixon AJ, Craven AJ, Pearon AJ (1995). The microanatomy, cell replication, and keratin gene expression of hair follicles during a photoperiod-induced growth cycle in sheep. Acta Anat.

[18] Pearson AJ, Parry AL, Ashby MG, Choy VJ, Wildermoth JE, Craven AJ (1996). Inhibitory effect of increased photoperiod on wool follicle growth. J Endocrinol.

[19] Celi P, Seren E, Celi R, Parmeggiani A, Di Trana A (2016). Relationships between blood hormonal concentrations and secondary fibre shedding in young cashmere-bearing goats at their first moult. Anim Sci.

[20] Shamsalddini S, Mohammadabadi MR, Esmailizadeh AK (2016). Polymorphism of the prolactin gene and its effect on fiber traits in goat. Genetika.

[21] Millar SE (2002). Molecular mechanisms regulating hair follicle development. J Invest Dermatol.

[22] Stenn KS, Combates NJ, Eilertsen KJ (1996). Hair follicle growth controls. Dermatol Clin.

[23] Kajdaniuk D, Marek B, Borgiel-Marek H, Kos-Kudła B (2011). Vascular endothelial growth factor (VEGF) — part 1: in physiology and pathophysiology. Endokrynol Pol.

[24] Lachgar S, Moukadiri H, Jonca F (1996). Vascular endothelial growth factor is an autocrine growth factor for hair dermal papilla cells. J Invest Dermatol.

[25] Yoon SY, Dieterich LC, Karaman S (2019). An important role of cutaneous lymphatic vessels in coordinating and promoting anagen hair follicle growth. PLOS ONE.

[26] Sha K, Chen M, Liu F (2020). Platelet factor 4 inhibits human hair follicle growth and promotes androgen receptor expression in human dermal papilla cells. PeerJ.

[27] Hyder SM, Stancel GM, Loose-Mitchell DS (1994). Steroid hormone-induced expression of oncogene encoded nuclear proteins. Crit Rev Eukaryot Gene Expr.

[28] Grichnik JM, Burch JA, Burchette J, Shea CR (1998). The SCF/kit pathway plays a critical role in the control of normal human melanocyte homeostasis. J Invest Dermatol.

[29] Randall VA, Jenner TJ, Hibberts NA, O De Oliveira I, Vafaee T (2008). Stem cell factor/C-kit signalling in normal and androgenetic alopecia hair follicles. J Endocrinol.

