# Supplementary material for: Effect of inhibiting prolactin secretion on secondary hair follicle development in cashmere goats
Source: Anim Biosci. 2025 May 12;38(11):2336–49. doi: 10.5713/ab.25.0053 (PMC12580954; doi:10.5713/ab.25.0053)
Supplement: Supplementary file 2 [file ab-25-0053-supplementary-2.pdf]

Supplement 2. RNA quality from cashmere goat skin

Sample	Concentration (ng/μL)	RIN	28S/18S	Total (μg)	Grade
T1	332	7.40	1.4	8.30	A
T2	410	8.10	1.8	10.25	A
T3	236	7.40	1.2	6.15	A
C1	278	5.80	1.3	9.73	A
C2	259	7.30	1.2	6.48	A
C3	200	6.80	1.4	5.00	A
