# Supplementary material for: Effect of inhibiting prolactin secretion on secondary hair follicle development in cashmere goats
Source: Anim Biosci. 2025 May 12;38(11):2336–49. doi: 10.5713/ab.25.0053 (PMC12580954; doi:10.5713/ab.25.0053)
Supplement: Supplementary file 3 [file ab-25-0053-supplementary-3.pdf]

Supplement 3. Statistics result of RNA-Seq reads comparison from skin

sample	Clean reads	Map reads	Percentage (%)	Error (%)	GC content (%)
T1	42997924	41462484	96.43	0.03	53.33
T2	44880710	43342479	96.57	0.02	52.54
T3	40534486	39036475	96.3	0.03	52.31
C1	44601110	42875802	96.13	0.02	52.48
C2	40972802	39573891	96.59	0.03	52.38
C3	44138942	42481851	96.25	0.03	52.36
