# Supplementary material for: Effect of inhibiting prolactin secretion on secondary hair follicle development in cashmere goats
Source: Anim Biosci. 2025 May 12;38(11):2336–49. doi: 10.5713/ab.25.0053 (PMC12580954; doi:10.5713/ab.25.0053)
Supplement: Supplementary file 4 [file ab-25-0053-supplementary-4.pdf]

Supplementa 4. Top 20 mRNAs expressed in skin samples

T			C		
**Gene ID**	**Gene name**	**Expression abundance**	**Gene ID**	**Gene name**	**Expression abundance**
ENSCHIG00000021319	COL1A1	461417.53	ENSCHIG00000021319	COL1A1	188829.14
ENSCHIG00000025647	COL1A2	414642.36	ENSCHIG00000025647	COL1A2	125519.74
novel.546	-	289877.80	ENSCHIG00000014749	KRT14	104705.24
ENSCHIG00000015120	-	145051.61	ENSCHIG00000010087	EEF1A1	103800.18
ENSCHIG00000022057	-	132756.03	ENSCHIG00000001082	KRT5	99823.28
ENSCHIG00000014749	KRT14	128076.53	novel.546	-	98792.94
ENSCHIG00000020972	SPARC	115375.78	ENSCHIG00000010632	KRT17	54801.55
ENSCHIG00000010087	EEF1A1	110205.79	ENSCHIG00000020972	SPARC	53179.97
ENSCHIG00000001082	KRT5	106064.05	ENSCHIG00000015120	-	49486.50
ENSCHIG00000015383	KRT25	99055.65	ENSCHIG00000022194	-	49021.21
ENSCHIG00000024470	KRT27	77793.69	ENSCHIG00000021305	GSN	47863.19
ENSCHIG00000021923	-	77558.75	ENSCHIG00000022057	-	45569.10
ENSCHIG00000005102	-	71012.16	ENSCHIG00000012421	-	45037.59
ENSCHIG00000025510	-	67273.41	ENSCHIG00000014215	-	42320.88
novel.888	-	66515.46	ENSCHIG00000013613	DSP	40884.53
ENSCHIG00000010632	KRT17	62218.01	ENSCHIG00000012599	-	40171.02
ENSCHIG00000018684	-	59402.62	ENSCHIG00000010613	CST6	40027.77
ENSCHIG00000022194	-	56672.81	ENSCHIG00000026676	ACTG1	37879.04
ENSCHIG00000012421	-	56350.57	ENSCHIG00000015249	AHNAK	36273.81
