# Supplementary material for: Effect of inhibiting prolactin secretion on secondary hair follicle development in cashmere goats
Source: Anim Biosci. 2025 May 12;38(11):2336–49. doi: 10.5713/ab.25.0053 (PMC12580954; doi:10.5713/ab.25.0053)
Supplement: Supplementary file 5 [file ab-25-0053-supplementary-5.pdf]

Supplement 5. Top 10 up- and downregulated differential genes from skin

Up	FDR	Down	FDR
ENSCHIG00000019118	4.33E-17	ENSCHIG00000025384	2.07E-12
ENSCHIG00000013076	3.80E-15	ENSCHIG00000012501	5.86E-10
ENSCHIG00000011844	3.80E-15	ENSCHIG00000011699	2.11E-07
ENSCHIG00000010301	3.80E-15	ENSCHIG00000013424	2.28E-07
ENSCHIG00000019424	4.29E-15	ENSCHIG00000015364	2.71E-07
ENSCHIG00000022295	4.85E-10	ENSCHIG00000021405	5.51E-07
ENSCHIG00000018257	1.69E-09	ENSCHIG00000004756	8.12E-07
ENSCHIG00000016842	4.55E-09	ENSCHIG00000013844	1.62E-06
novel.1312	4.98E-08	ENSCHIG00000011048	2.85E-06
novel.888	6.60E-08	ENSCHIG00000024104	2.89E-06
