# Supplementary material for: Effect of inhibiting prolactin secretion on secondary hair follicle development in cashmere goats
Source: Anim Biosci. 2025 May 12;38(11):2336–49. doi: 10.5713/ab.25.0053 (PMC12580954; doi:10.5713/ab.25.0053)
Supplement: Supplementary file 6 [file ab-25-0053-supplementary-6.pdf]

Supplement 6. Top 20 pathways of KEGG enrichment

KEGG ID	Description	GeneRatio	BgRatio	*P*-value	FDR	Up	Down
chx04915	Estrogen signaling pathway	23/275	138/7477	8.38E-10	2.19E-07	13	10
chx05150	Staphylococcus aureus infection	11/275	81/7477	0.000167	0.021927	11	0
chx04010	MAPK signaling pathway	23/275	289/7477	0.00038	0.033152	1	22
chx04928	Parathyroid hormone synthesis, secretion, and action	12/275	107/7477	0.000528	0.034588	1	11
chx04810	Regulation of actin cytoskeleton	17/275	220/7477	0.00298	0.134358	1	16
chx04151	PI3K-Akt signaling pathway	24/275	361/7477	0.003443	0.134358	1	23
chx05163	Human cytomegalovirus infection	17/275	224/7477	0.00359	0.134358	1	16
chx04014	Ras signaling pathway	17/275	232/7477	0.005119	0.137965	2	15
chx04371	Apelin signaling pathway	13/275	160/7477	0.005933	0.137965	1	12
chx04510	Focal adhesion	15/275	200/7477	0.006766	0.137965	0	15
chx05220	Chronic myeloid leukemia	8/275	77/7477	0.007062	0.137965	0	8
chx05166	Human T-cell leukemia virus 1 infection	16/275	223/7477	0.007944	0.137965	0	16
chx01522	Endocrine resistance	9/275	95/7477	0.007989	0.137965	0	9
chx04213	Longevity regulating pathway - multiple species	7/275	63/7477	0.00802	0.137965	1	6
chx04926	Relaxin signaling pathway	11/275	130/7477	0.008205	0.137965	1	10
chx05206	MicroRNAs in cancer	13/275	167/7477	0.008425	0.137965	2	11
chx05231	Choline metabolism in cancer	9/275	99/7477	0.010369	0.159618	0	9
chx04512	ECM-receptor interaction	8/275	83/7477	0.010966	0.159618	0	8
chx05224	Breast cancer	11/275	139/7477	0.013209	0.182149	1	10
