# Supplementary material for: Effect of inhibiting prolactin secretion on secondary hair follicle development in cashmere goats
Source: Anim Biosci. 2025 May 12;38(11):2336–49. doi: 10.5713/ab.25.0053 (PMC12580954; doi:10.5713/ab.25.0053)
Supplement: Supplementary file 7 [file ab-25-0053-supplementary-7.pdf]

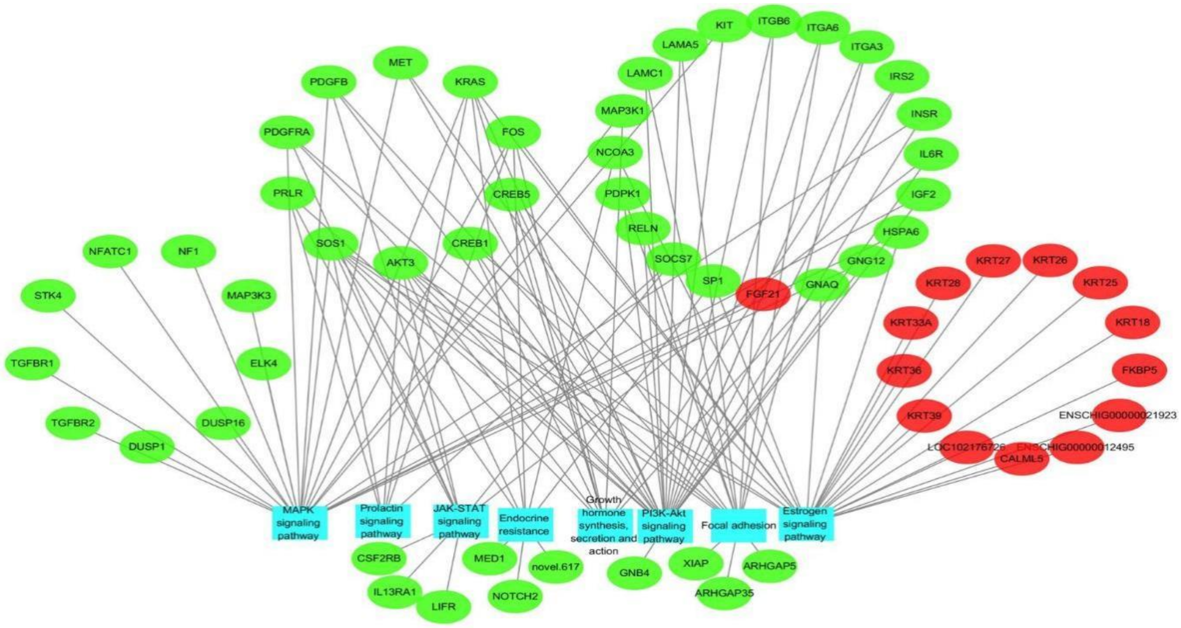

Supplement 7. KEGG enrichment of DEGs from skin. Gene network regulation of KEGG associated with HF development. Boxes represent pathways, circles represent genes involved in pathways, red represents genes upregulated in the test group, and green represents genes downregulated in the treatment group.
