# Supplementary figures and images for: Effect of inhibiting prolactin secretion on secondary hair follicle development in cashmere goats
Source: Anim Biosci. 2025 May 12;38(11):2336–49. doi: 10.5713/ab.25.0053 (PMC12580954; doi:10.5713/ab.25.0053)

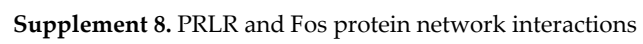

### Supplement 8. PRLR and Fos protein network interactions

Supplement: Supplementary file 8 [file ab-25-0053-supplementary-8.pdf]

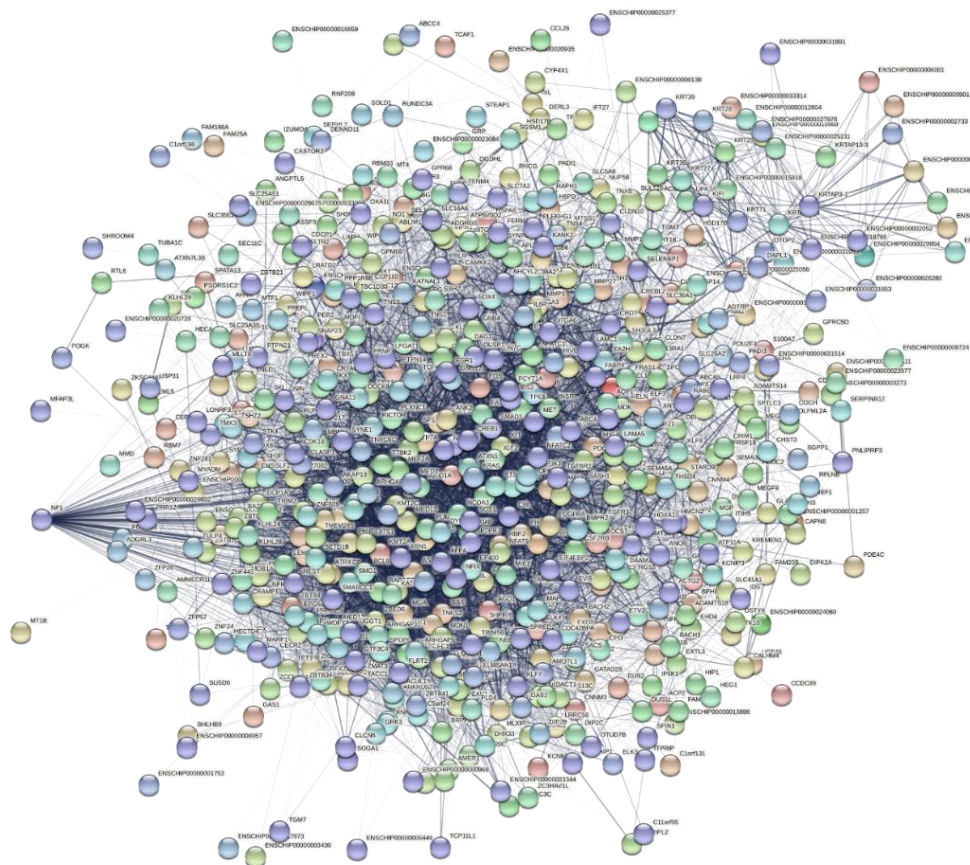

**Supplement 10.** Protein network interactions of DEGs from skin in cashmere non-growing period

Supplement: Supplementary file 10 [file ab-25-0053-supplementary-10.pdf]
