# Supplementary material for: Effect of inhibiting prolactin secretion on secondary hair follicle development in cashmere goats
Source: Anim Biosci. 2025 May 12;38(11):2336–49. doi: 10.5713/ab.25.0053 (PMC12580954; doi:10.5713/ab.25.0053)
Supplement: Supplementary file 9 [file ab-25-0053-supplementary-9.pdf]

Supplement 9. DEG information for interactions with PRLR and Fos proteins

Protein	Interaction score with PRLR	Interaction score with Fos
PDGFB	0.949	0.289
IRS2	0.612	0.337
IL6R	0.545	0.164
IGF2	0.417	0.423
TP63	0.253	0.169
ETS1	0.248	0.838
PTGS2	0.235	0.704
LIFR	0.227	0.203
RHCG	0.223	0.401
PPP1R9B	0.205	0.166
VIPR1	0.204	0.233
MT4	0.202	0.234
ACHE	0.201	0.333
Kit	0.184	0.331
ATF3	0.181	0.905
NR3C1	0.173	0.914
PRNP	0.163	0.164
