# Supplementary material for: Effect of inhibiting prolactin secretion on secondary hair follicle development in cashmere goats
Source: Anim Biosci. 2025 May 12;38(11):2336–49. doi: 10.5713/ab.25.0053 (PMC12580954; doi:10.5713/ab.25.0053)
Supplement: Supplementary file 11 [file ab-25-0053-supplementary-11.pdf]

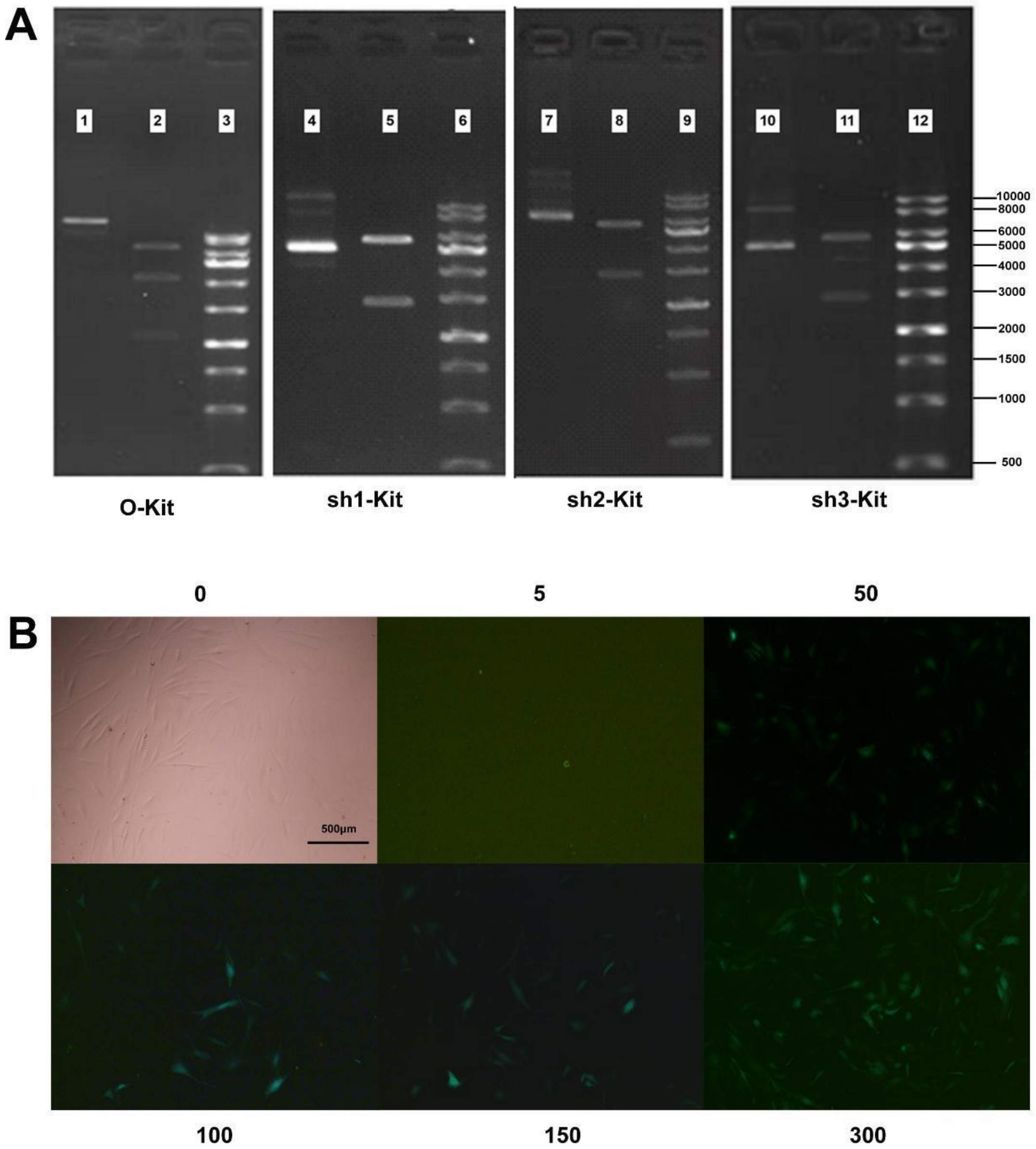

Supplementary 11. Screening for optimal MOI value for infected DPCs. (A) 1% gel electrophoresis，o-Kit (1–3) plasmid identification by ScaI and NheI enzyme digestion, sh1-Kit (4–6), sh2-Kit (7–9), and sh3-Kit (10–12) plasmid identification by AflII enzyme digestion. (B) Brightfield DPC morphology (o), Fluorescence of encapsulated empty plasmid lentivirus-infected DPCs with different MOI values (5, 50, 100, 150, and 300).
