# Supplementary material for: Effect of inhibiting prolactin secretion on secondary hair follicle development in cashmere goats
Source: Anim Biosci. 2025 May 12;38(11):2336–49. doi: 10.5713/ab.25.0053 (PMC12580954; doi:10.5713/ab.25.0053)
Supplement: Supplementary file 1 [file ab-25-0053-supplementary-1.pdf]

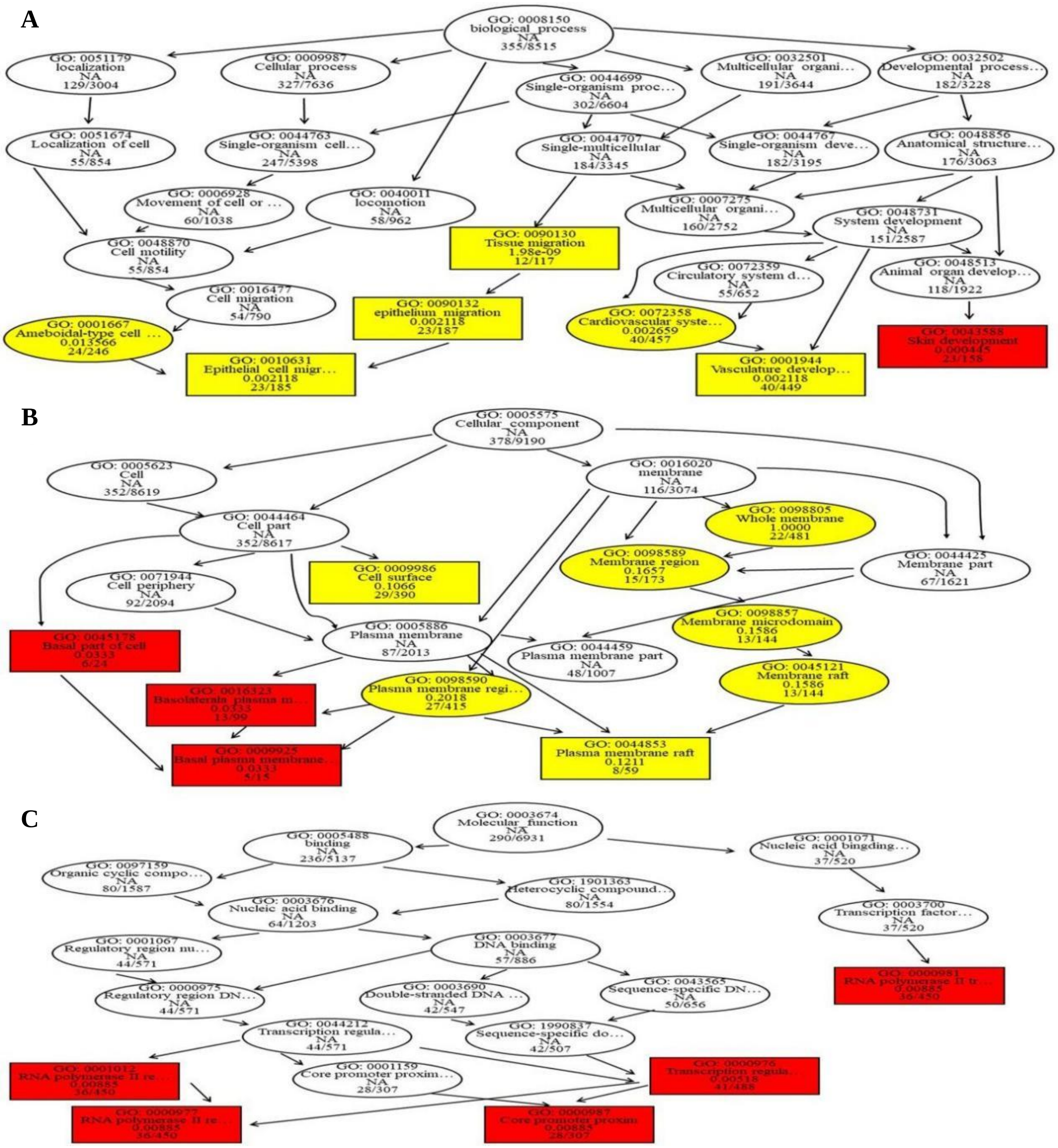

Supplement 1. GO enrichment of DEGs from skin. (A) DAG plot of GO terms (BP class). (B) DAG plot of GO terms (CC class). (C) DAG plot of GO terms (MF class).
